# Altered network stability in progressive supranuclear palsy

**DOI:** 10.1016/j.neurobiolaging.2021.07.007

**Published:** 2021-11

**Authors:** David J Whiteside, P. Simon Jones, Boyd C P Ghosh, Ian Coyle-Gilchrist, Alexander Gerhard, Michele T. Hu, Johannes C Klein, P. Nigel Leigh, Alistair Church, David J Burn, Huw R Morris, James B Rowe, Timothy Rittman

**Affiliations:** a Cambridge University Department of Clinical Neurosciences and Cambridge University Hospitals NHS Trust, University of Cambridge, UK; b Wessex Neurological Centre, University Hospital Southampton, Southampton, UK; c Norfolk and Norwich University Hospital, Norwich, UK; d Division of Neuroscience and Experimental Psychology, University of Manchester, Manchester, UK; e Oxford Parkinson's Disease Centre and Nuffield Department of Clinical Neurosciences, University of Oxford, Oxford, UK; f Department of Neuroscience, Brighton and Sussex Medical School, Brighton, UK; g Department of Neurology, Royal Gwent Hospital, Newport, UK; h Faculty of Medical Sciences, Newcastle University, Newcastle, UK; i Department of Clinical and Movement Neurosciences, University College London. Queen Square Institute of Neurology, London, UK

**Keywords:** Progressive supranuclear palsy, Network dynamics, Hidden Markov models, Complexity, PSP, Progressive supranuclear palsy, HMM, Hidden Markov models, MSE, Multi-scale entropy, ICA, Independent component analysis, PCA, Principal component analysis, PSPRS, Progressive supranuclear palsy-rating-scale, ACE, Addenbrooke's Cognitive Examination, CCPP, Cambridge Centre for Parkinson-plus, PROSPECT-MR, Progressive Supranuclear Palsy-Corticobasal Syndrome-Multiple System Atrophy-UK study

## Abstract

The clinical syndromes of Progressive Supranuclear Palsy (PSP) may be mediated by abnormal temporal dynamics of brain networks, due to the impact of atrophy, synapse loss and neurotransmitter deficits. We tested the hypothesis that alterations in signal complexity in neural networks influence short-latency state transitions. Ninety-four participants with PSP and 64 healthy controls were recruited from two independent cohorts. All participants underwent clinical and neuropsychological testing and resting-state functional MRI. Network dynamics were assessed using hidden Markov models and neural signal complexity measured in terms of multiscale entropy. In both cohorts, PSP *increased* the proportion of time in networks associated with higher cognitive functions. This effect correlated with clinical severity as measured by the PSP-rating-scale, and with reduced neural signal complexity. Regional atrophy influenced abnormal brain-state occupancy, but abnormal network topology and dynamics were not restricted to areas of atrophy. Our findings show that the pathology of PSP causes clinically relevant changes in neural temporal dynamics, leading to a greater proportion of time in inefficient brain-states.

## Highlights

• We investigated network dynamics in the tauopathy progressive supranuclear palsy
• Abnormal temporal properties of large-scale networks are related to phenotype
• Progressive supranuclear palsy paradoxically *increases* frontoparietal state time
• Reductions in neural signal complexity relate to altered network dynamics
• Dynamic network and topological changes occur distally to primary sites of atrophy

## 1 Introduction

The human brain optimises efficiency by balancing integration and segregation of information transfer among neural assemblies. The activity and connectivity of regional specialisation is dynamic (Deco et al., 2017; Friston et al., 2012; Honey et al., 2007; Shine et al., 2019; Tognoli and Kelso, 2014), even on the suprasecond timescale of functional magnetic resonance imaging (Calhoun et al., 2014; Hindriks et al., 2016; Vidaurre et al., 2017). The co-ordination of such state transitions depends on the divergent topological properties of cortical and subcortical regions (Gu et al., 2015), and may be moderated by the principal inhibitory and excitatory neurotransmitters, GABA and glutamate. In the neurodegenerative tauopathies, the pattern of spread of tau-pathology is dictated in part by the brain's topology and connectivity (Ahmed et al., 2014; Clavaguera et al., 2009; Seeley et al., 2009), leading to reductions in effective information processing and cognition.

We proposed that alterations in large-scale network dynamics contribute to the severity of neurodegenerative syndromes. We test this using the tau-associated disease progressive supranuclear palsy (PSP), as a demonstrator condition because of its high clinicopathological correlation. To quantify signal complexity and network dynamics we use task-free functional MRI (fMRI) from two independent cohorts.

The clinical features of PSP, together with its established imaging and pathological findings, qualify it as a model disease to investigate network dynamics. PSP has prominent cognitive and behavioral features, including a dysexecutive frontal syndrome, apathy, impulsivity and language impairment, in addition to the movement disorder of axial akinetic-rigidity and impaired postural reflexes (Burrell et al., 2014; Steele et al., 1964). The disruption to static functional connectivity in PSP (Brown et al., 2017; Gardner et al., 2013; Rosskopf et al., 2017; Whitwell et al., 2011) affects frontal cortical regions associated with cognitive control and behavior, alongside striatal degeneration and loss of dopaminergic and noradrenergic projections from the brainstem to forebrain (Murley and Rowe, 2018). The latter are critical to balance network integration and segregation (Shine, 2019), with catecholaminergic deficits related to dynamic connectivity, cognitive performance and disease severity (Eldar et al., 2013; Kaalund et al., 2020; Shine et al., 2018).

Network dynamics need to be interpreted in the context of neural complexity (Honey et al., 2007; McDonough and Nashiro, 2014). Complexity varies with the timescale analyzed, with the potential for scale dependent relationships between integrative or synchronous activity, state switching and complexity (McDonough and Nashiro, 2014; McIntosh et al., 2014; Wang et al., 2018). This relationship may be due to interference between neural complexity and regional phase relationships, decreasing the likelihood of synchrony between brain regions (Ghanbari et al., 2015). Alternatively, sufficient signal complexity may be required to establish long range dependencies, leading to a positive relationship between connectivity and complexity conditional on timescale (McDonough and Nashiro, 2014; Wang et al., 2018).

Entropy measures can assess complexity in the relatively short, non-linear and noisy time series typical of fMRI (Grandy et al., 2016; Pincus and Goldberger, 1994; Turkheimer et al., 2015). Sample entropy measures the likelihood that repeated patterns are present in data: signals with a repetitive structure have lower entropy (Richman and Moorman, 2000). Multiscale entropy (MSE) extends sample entropy by assessment at multiple timescales, with the advantage that random noise can be differentiated from complex signal: random fluctuations increase entropy at fine time scales, but with increasing the timescale entropy decreases (Costa et al., 2005).

Large-scale network dynamics can be quantified by hidden Markov modelling (HMM), in terms of a finite number of mutually exclusive states between which the brain switches over time (Vidaurre et al., 2017). While states are inferred at a group level, information is obtained about the order and timing of individuals’ state transitions.

We used these methods to investigate the impact of PSP on network dynamics, as a function of changes in signal complexity, brain structure and functional reorganisation. We examined two contemporary but independent cohorts of PSP, and controls, from the Cambridge Centre for Parkinson-plus (CCPP) and the UK national PSP Research Network (PROSPECT-MR). In each cohort, we analyzed HMM and MSE of task-free functional magnetic resonance. We then tested whether the network properties in PSP varied as a function of disease severity (PSP-rating-scale) and PSP phenotype (Richardson's syndrome, cortical- and sub-cortical variants).

## 2 Methods

### 2.1 Participants

Forty-five participants with PSP (possible or probable, according to MDS-PSP criteria (Höglinger et al., 2017)) and 27 controls were recruited at the Cambridge University Centre for Parkinson-plus (CCPP). 49 study participants with PSP and 37 controls were recruited to Progressive Supranuclear Palsy-Corticobasal Syndrome-Multiple System Atrophy-UK (PROSPECT-MR) study (Jabbari et al., 2019). 34 participants (28 PSP, 6 controls) were removed following assessment of motion and image quality (section 2.2). For both cohorts clinical assessment included the PSP-rating-scale (PSPRS) (Golbe and Ohman-Strickland, 2007) and Addenbrooke's Cognitive Examination (ACE: Addenbrooke's Cognitive Examination-Revised for CCPP (Mioshi et al., 2006), Addenbrooke's Cognitive Examination-III for PROSPECT-MR (Hsieh et al., 2013)). Summary scores and demographic details are outlined in Table 1.

**Table 1:** Demographic and clinical characteristics of study participants

	CCPP: Control	CCPP: PSP	t/ χ (p)	PROSPECT: Control	PROSPECT: PSP	t/ χ (p)
Number	22	24		36	42	
Age (years)	64.9 (9.9)	70.1 (6.5)	t=2.1 (*p*=0.038)	67.3 (7.1)	71.1 (7.3)	t=2.4 (*p*=0.021)
Gender (F/M)	14/8	11/13	χ =1.5 (*p*=0.23)	26/10	15/27	χ =10.4 (*p*=0.001)
PSP clinical phenotype (n)		PSP-RS = 16 PSP-subcortical= 0 PSP-cortical=8			PSP-RS = 25 PSP-subcortical= 11 PSP-cortical=6	
ACE		82 (11.4)		95.7 (3.4)	81.3 (11.6)	t=6.9 (*p*<0.0001)
PSPRS		34.9 (12.5)			33.9 (14.2)	

Continuous values are mean (SD). Group comparison used *t*-test for groups with continuous data and chi-squared for binary variables. (PSP-RS: PSP-Richardson syndrome, ACE: Addenbrooke's Cognitive Examination, PSPRS: Progressive Supranuclear Palsy-Rating-Scale)

PSP is a heterogeneous syndrome with variant presentations other than the classical Richardson's syndrome (Jabbari et al., 2019). The clinical phenotype is related to the distribution of tau and focal grey matter loss, allowing us to test whether variation in the topographical distribution of disease burden (Ling et al., 2014; Sakae et al., 2019; Tsuboi et al., 2005) or atrophy (Jabbari et al., 2019) results in distinct changes in network dynamics. In keeping with Jabbari et al, clinical phenotype was categorized as PSP Richardson's syndrome (PSP-RS), PSP-subcortical (i.e., PSP-P with predominant parkinsonism or PSP-PGF with progressive gait freezing) or PSP-cortical (PSP-F with frontal presentations, PSP-CBS with corticobasal features or other focal cortical syndromes) (Jabbari et al., 2019). Clinical phenotypes for both cohorts are included in Table 1.

### 2.2 MRI acquisition and preprocessing

Participants at CCPP underwent fMRI imaging at 3T using echo-planar imaging sensitive to the blood-oxygen-level-dependent signal (TR 2 secs, TE 30ms, whole brain acquisition, 3 × 3 × 3.75mm voxels, 305 volumes) with eyes open in a dark bore. High resolution T1-weighted Magnetization Prepared Rapid Gradient Echo (MPRAGE) structural images (TR 2s, TE 2.93ms, flip angle 8°, voxel size 1.1mm isotropic) were acquired during the same session for use in normalization. Participants from PROSPECT-MR underwent a comparable fMRI imaging protocol at 3T (TR 2.5 secs, TE 30ms, whole brain acquisition, 3 × 3 × 3.5mm voxels, 200 volumes) and matched MPRAGE.

Image preprocessing used FSL's FEAT for registration to the structural image, motion correction, 100Hz high-pass temporal filtering, 5mm FWHM spatial smoothing. Denoising was performed using FSL's FIX with a training set of 10 subjects from each disease group per cohort. Additional removal of motion artefact used wavelet despiking (brainwavelet.org).

Given the sensitivity of estimates of network dynamics to participant motion (Laumann et al., 2017; Nickerson et al., 2017; Power et al., 2012), we excluded thirty-three participants (27 PSP, 6 Control) with greater than 1 standard deviation from the whole sample mean for any of four data quality indices obtained from an fMRI dataset of 408 controls or participants with neurodegenerative diseases (maximum spike percentage (Patel et al., 2014), median spike percentage, maximum framewise displacement (Power et al., 2012), maximum spatial standard deviation of successive volume difference (Smyser et al., 2010)), and one participant with PSP for incomplete data. Summary measures by group are in supplementary table 1. We took the average of the four metrics post standardization as a covariate of no interest in further analyses.

### 2.3 Structural MRI

We extracted cortical thickness and subcortical grey matter volumes for 246 nodes of the Brainnetome Atlas (Fan et al., 2016) and volumes for four brainstem substructures (midbrain, pons, medulla and superior cerebellar peduncle) (Iglesias et al., 2015) using Freesurfer 6.0 (Dale et al., 1999). We compared differences between participants with PSP and controls in thicknesses and volumes with permutation testing, family-wise error correction for multiple comparisons, and a statistical threshold of *p*<0.05. Age and total intracranial volume were included as nuisance variables. We compared network dynamic metrics with atrophy measures, focusing on subcortical volumes and frontal cortical thicknesses given that neuropathological changes occur earlier and sequentially in these regions (Kovacs et al., 2020).

### 2.4 Hidden Markov modelling

To investigate changes in network dynamics in PSP we first performed a group independent component analysis using FSL's MELODIC tool on preprocessed fMRI from controls and participants with PSP. We chose a model order of 30 as a balance between excessive network fragmentation (Ray et al., 2013) and predetermining HMM outputs. The same component maps were used for both datasets. Participant specific timecourses for each component were generated by the first stage of a dual regression. From standardized per participant component timecourses a multivariate Gaussian HMM with 8 brain states was inferred using the HMM-MAR toolbox (Vidaurre et al., 2017). A common covariance matrix was modelled for all states, so that the dynamic structure was driven primarily by activation changes. This approach was adopted to ensure that the model would effectively capture network dynamics in a cohort where there may be significant between-subject differences in static connectivity (see (Vidaurre et al., 2021) for a summary of different possible approaches to modelling with HMM). The order selection in an HMM is predetermined; it has previously been shown that 8 states capturing large scale networks can provide robust behavioral inferences (Vidaurre et al., 2018).

Different metrics can be used to quantify the temporal characteristics of the HMM states, including *switching rate* (the frequency with which states transitions occur) and *fractional occupancy* (the proportion of time a state is active). We assessed between-group differences in these metrics. Given the interdependence of fractional occupancy rates we performed a principal component analysis (PCA) to compare with severity measures.

### 2.5 Multiscale entropy

To investigate changes in complexity we calculated MSE for the same component timeseries used to infer the HMM, adapting LOFT's Complexity toolkit (Smith et al., 2014). We averaged over a fixed number of timescales (3 scales PROSPECT-MR, 4 for CCPP due to the longer time series), and calculated sample entropy on the time series constructed for each scale (Costa et al., 2005). MSE is then sum of sample entropy across all timescales. We selected pattern length of 1 and pattern matching threshold of 0.35 given evidence that these parameters provide robust results (Yang et al., 2018). We took the average MSE calculated across the 30 component timeseries for further analyses. We assessed between-group differences and the correlation across-subjects between complexity and fractional occupancy.

### 2.6 Graph measures

We performed graph theoretical analysis using Maybrain software (https://github.com/RittmanResearch/maybrain) and NetworkX (Hagberg et al., 2008), with the Brainnetome parcellation. Association matrices were constructed by taking the wavelet cross-correlation between each region using a maximal overlap discrete wavelet transform and Daubechies filter performed using the waveslim package in R. The second band of 4 was used corresponding to a frequency range of 0.0675-0.125Hz (Achard and Bullmore, 2007).

To test our hypothesis that changes in network dynamics were related cortical network topological remodelling in PSP in response to subcortical tau burden, we focused on the following metrics: *weighted degree*, measuring the number and strength of nodal functional connections; *clustering coefficient*, the proportion of triangular connections formed by each node over the proportion of all possible such connections; and *path length*, the average shortest topological distance between nodes of the graph. The combination of path length and clustering coefficient defines randomness or regularity of the graph, with random graphs exhibiting short path length and small clustering coefficient (Watts and Strogatz, 1998). Path length and clustering coefficient were assessed across the brain, and weighted degree in cortical and subcortical regions and between groups. Metrics except for weighted degree were binarised after thresholding and normalised against 1000 random graphs with identical degree distribution and random connections. A network density threshold of 5% was used. We also report results at density thresholds of 1%–10% for significant results to ensure robustness.

### 2.7 Statistical approach

We conducted initial analysis in the two cohorts separately. This was to allow analysis of MSE at higher scales in the CCPP cohort, thereby increasing our ability to differentiate complex signal from randomness and to contrast with HMM metrics, given that with fewer than 50 timepoints error of sample entropy estimates may increase (Yang et al., 2013; Yang et al., 2018). Replication can ensure that results are robust, an important factor given concerns that apparent changes in network dynamics from resting state fMRI are attributable to analysis techniques, head motion and sleep (Laumann et al., 2017).

Statistical tests used a general linear model with permutation testing (10000 permutations), with family-wise error correction for multiple comparisons and contrasts using FSL's PALM (Winkler et al., 2014) and a statistical threshold of *p*<0.05. The exceptions were moderation analysis, comparisons with graph metrics and direct tests of slope, which were performed in R (R Core Team, 2018). Participant motion, age and sex (estimated total intracranial volume for contrasts involving measures of volume) were included as nuisance variables.

Given demographic differences in both cohorts we repeated our analysis on a subset of participants, using the *matchit* function in R to select participants. We initially removed participants outside the common support region, followed by nearest neighbour matching with a caliper of 0.5 if groups remained unbalanced.

## 3 Results

### 3.1 Demographics

We present analyses from 24 participants with PSP and 22 controls from CCPP, and 42 participants with PSP and 36 controls from PROSPECT-MR. Demographic details are outlined in Table 1. There were significant differences in age in both cohorts and in gender in PROSPECT-MR.

### 3.2 Network dynamics

**Fig. 1:**
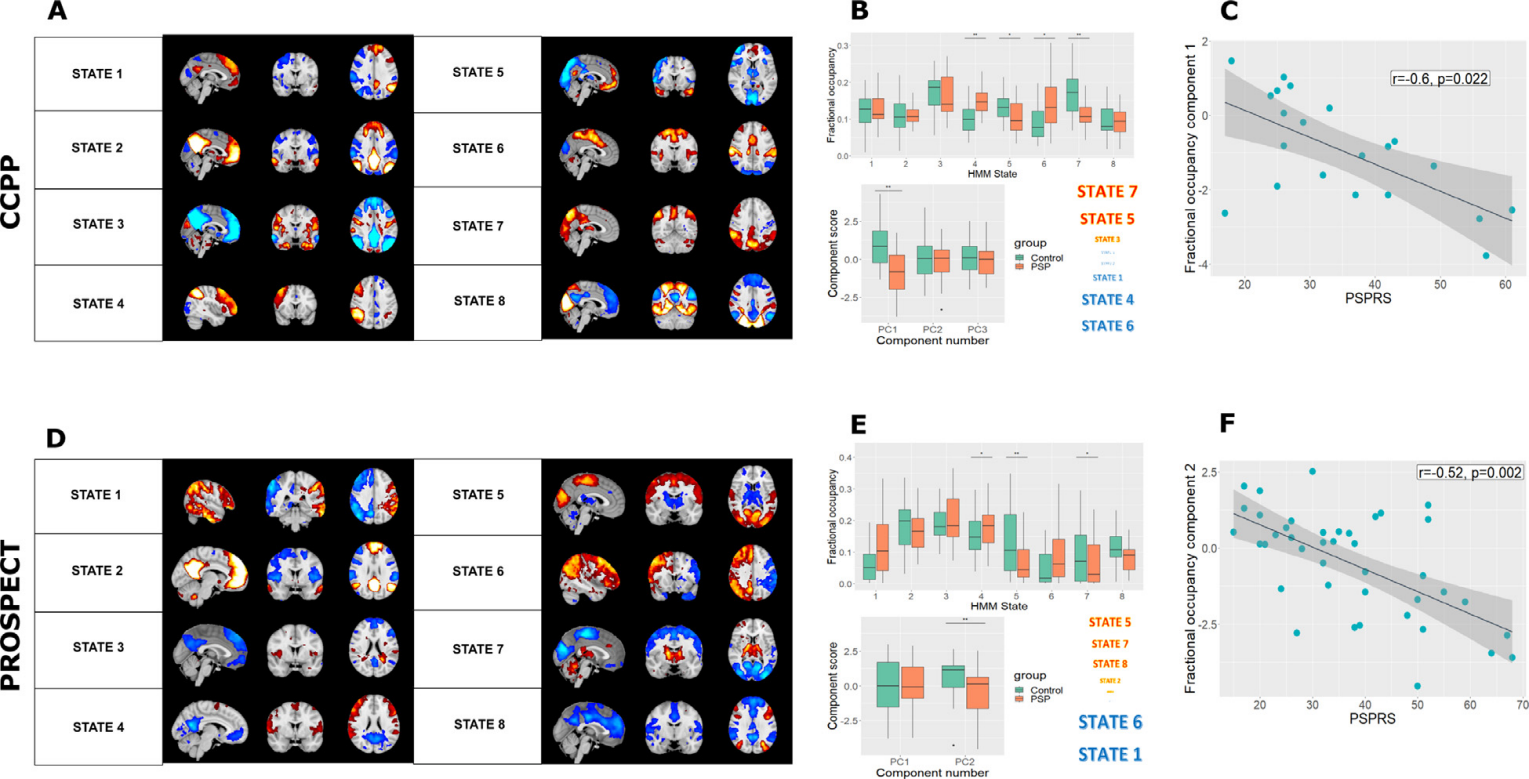
Network dynamics in PSP vs controls. (A) and (D) Mean activation maps for the 8 inferred brain states from hidden Markov modelling for the two cohorts. (B) and (E) Altered fractional occupancy rates in PSP for the two cohorts. Results are shown both by differences in states computed within a general linear model with a single permutation test and family-wise error correction, and in a principal component analysis of fractional occupancy rates. In both cohorts, participants with PSP spent less time in states with subcortical and posterior activation, and more time in frontoparietal states. Colours of state names indicate direction of principal component loading, and font size scales with their magnitude. (C) and (F) The component that differed between PSP and controls correlated with PSP rating scale among patients (CCPP r=-0.6, *p*=0.02; PROSPECT r=-0.52, *p*=0.002).

CCPP: We used temporally concatenated participant timeseries from ICA components to fit an HMM with 8 brain states. Mean activation maps for these states are shown in Fig. 1A.

There was no difference in switching rate between controls and participants with PSP (t=0.37, *p*=0.59). We performed a PCA of fractional occupancy rates, which are collinear and compositional. Three components with eigenvalues greater than 1 explained 75% of the variance and were taken forward for further analysis. The first component was significantly more negative in PSP than controls (t=4.0, *p*=0.0008) (Fig. 1B). States with the highest positive loadings (states 5 and 7) had prominent subcortical and posterior activation, while states with the most negative loadings (states 1, 4 and 6) largely constituted the executive control and salience networks.

PROSPECT-MR: Mean activation maps for the 8 HMM inferred PROSPECT-MR brain states are shown in Fig. 1D. States 1-4 were the closest Dice coefficient matches (supplementary figure 1) for both positive and negative maps. There were anatomical differences in the mean activation maps between the two cohorts, particularly in identified anti-correlations, resulting in divergence between designations for positive and negative maps. Therefore, we adjudicated matching for the remaining states by visual inspection.

There was no difference in switching rates between the two groups, but participants with PSP had altered fractional occupancy (see Fig. 1E). Two components with eigenvalues greater than 1 explained 74% of the variance and were taken forward for further analysis. Component scores for the second component were significantly decreased in PSP (t=3.1, *p*=0.006). States with the most positive loadings (states 5 and 7) had prominent subcortical, posterior and motor region activations, while states with negative loadings overlapped with executive networks (states 1 and 6).

Given the demographic differences in both cohorts we repeated our analysis using a subset of participants matched for age and sex (see supplementary material). Our key findings were unchanged in both cohorts.

In summary, in both groups participants with PSP spent a greater proportion of time in states of activity in executive networks, and away from states of activity in networks with posterior and subcortical activations.

### 3.3 Complexity

We investigated whether complexity differed between PSP and Controls, to provide further insight into temporal dynamics in the disease.

**Fig. 2:**
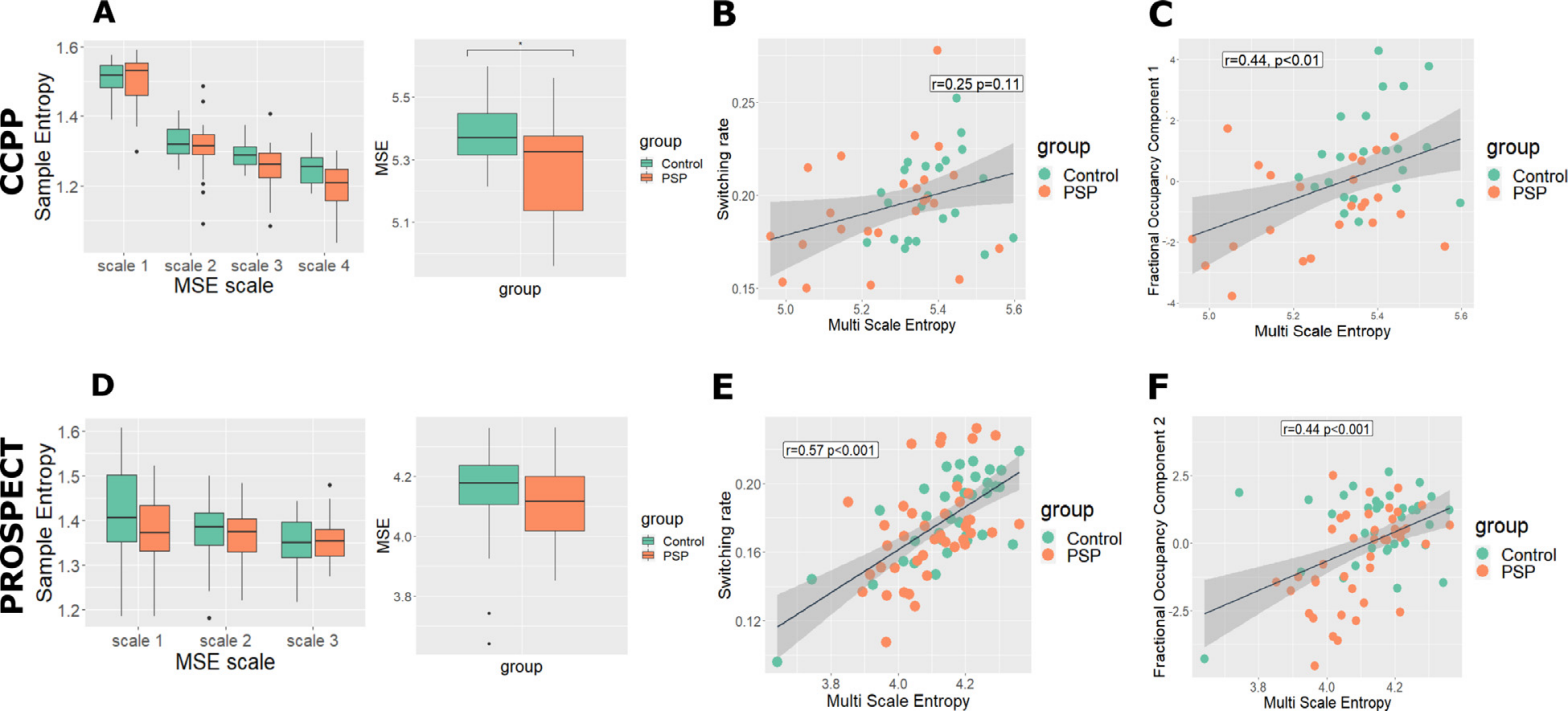
Complexity analysis. (A) and (D) We found complexity to be reduced in PSP in the CCPP, but not in PROSPECT-MR. (B) and (E) In PROSPECT-MR but not CCPP multiscale entropy (MSE) correlated significantly with switching rate. (C) and (F) MSE correlated with the fractional occupancy component that differed between PSP and controls in CCPP.

CCPP: We calculated MSE for each participant at 4 timescales using the same component timeseries used to infer the HMM. MSE was significantly reduced in PSP (t=2.3, *p*=0.022, Fig. 2A).

PROSPECT-MR: MSE was calculated over 3 rather than 4 timescales, due to shorter time series. In contrast to our locally collected data, we did not find the reduction in MSE in PSP to be significant (t=1.1, *p*=0.27, Fig. 2D)

### 3.4 Network dynamics and clinical severity

We tested whether the distinct changes in network dynamics in PSP were related to clinical severity, as measured by the PSPRS and ACE.

CCPP: The first fractional occupancy component negatively correlated with the PSPRS (r=-0.60, *t*=-3.1, *p*=0.022, see Fig. 1C). There was also a relationship between principal component 3 and ACE which was not significant after correction for multiple comparisons (r=0.47, t=2.4, uncorrected *p*=0.03).

PROSPECT-MR: Component scores for the second fractional occupancy component correlated with PSPRS (r=-0.52, t=-3.7, *p*=0.002, Fig. 1F). There was also a relationship between the second fractional occupancy component and ACE which was not significant after correction for multiple comparisons (r=-0.34, t=-2.1, uncorrected *p*=0.046).

### 3.5 Network dynamics and complexity

We investigated the relationship between signal complexity and network dynamics. We asked whether a) the distinct changes in fractional occupancy were related to complexity and b) switching rate related to complexity, and whether these relationships interacted with diagnosis.

*3.5.1 Fractional occupancy and MSE*. In both groups there was a significant relationship between MSE and the fractional occupancy component that differed between people with PSP and Controls (CCPP r=0.44, t=3.2, *p*=0.004, Fig. 2C; PROSPECT-MR r=0.44, t=4.2, *p*=0.0003, Fig. 2F). Moderation analysis did not show any significant group differences in these relationships. In PROSPECT-MR the slope in controls was driven by a single outlier; post outlier removal the relationship between component and complexity differed by diagnosis (PROSPECT-MR Δr^2^ = 0.08, F=9.8, *p*=0.003).

*3.5.2 Switching rates and MSE:* In the CCPP group MSE did not correlate significantly (Figs. 2B) with switching rate (r=0.25, t=1.6, *p*=0.11). In the PROSPECT-MR group a significant relationship was found (r=0.57, t=6, *p*=0.0001, Fig. 2E). Moderation analysis did not show any significant group differences in these relationships.

### 3.6 Network dynamics in PSP versus structure, topology, and clinical presentation

We tested the hypothesis that people with PSP spend a greater proportion of time in inefficient states due to cortical remodelling in response to focal disease. Given that distribution of atrophy and pathology differ by phenotype, we tested whether network dynamics vary by clinical phenotype. Since the two datasets showed overlapping changes in brain state occupancy in PSP, we performed a combined analysis to investigate these hypotheses, focusing on fractional occupancy. We assessed whether fractional occupancy components: (i) relate to frontal cortical thickness and subcortical volumes; (ii) relate to regional topological remodeling; and iii) differed depending on PSP clinical phenotype.

**Fig. 3:**
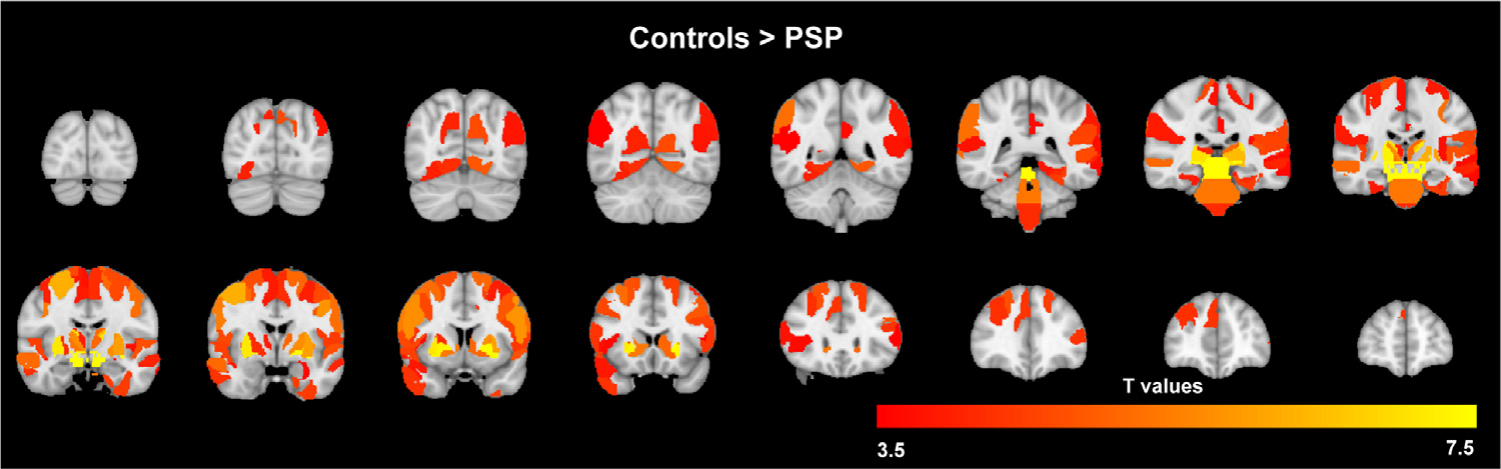
Significant areas of grey matter volume reduction in PSP v controls in a combined analysis across the two cohorts, where regions are nodes of the Brainnetome Parcellation. *p*<0.05 after family-wise error correction for multiple comparisons. There were no regional differences in direct comparison of participants with PSP from the two cohorts.

We first sought to investigate the distribution of atrophy across the two cohorts. For participants with PSP we found significant areas of grey matter atrophy compared to controls in the midbrain and subcortical regions defined by the Brainnetome Atlas (Fig. 3). We also found cortical atrophy primarily in the frontal lobe and peri-Rolandic regions, but also in the temporal and parietal cortex. There were no regional differences when comparing participants with PSP from the two cohorts.

**Fig. 4:**
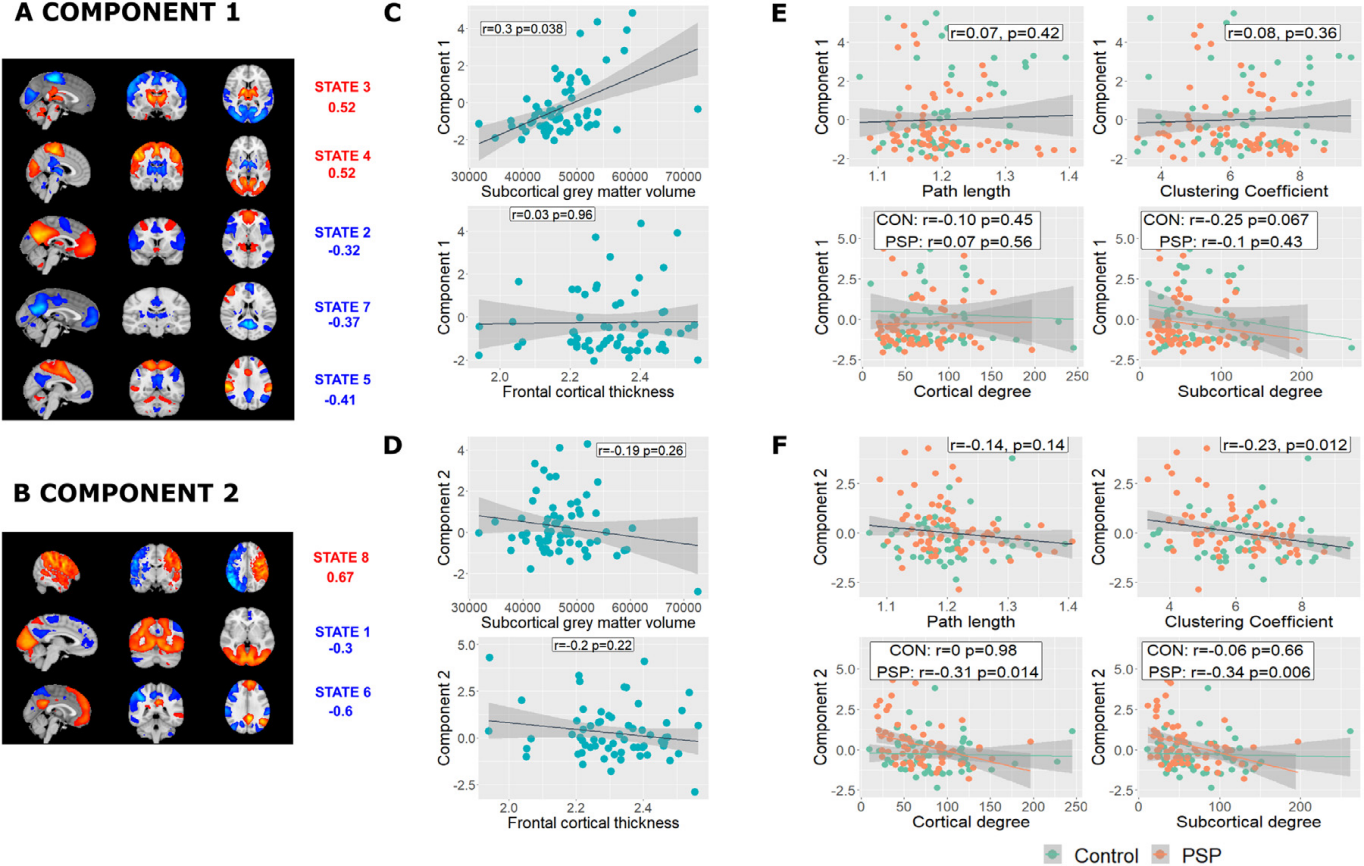
Network dynamics, atrophy and network topology. (A) and (B) In a combined analysis of the two cohorts the first two principal components differed between PSP and controls. Mean activation states with PCA loadings >|0.3| are shown. (C) and (D) Component 1 correlated with subcortical volume but not frontal cortical thickness, although with no significant difference in slope. Component 2 did not correlate significantly with either frontal cortical thickness or subcortical volume. E and F) Loadings in component 2 were associated with reduced clustering coefficient and reduced weighted degree in PSP but not controls. No relationships were found with component 1.

The two principal components derived from HMM analysis (Figs. 4A and 4D) were taken forward for analysis differed between PSP and controls (component 1 t=2.6, *p*=0.023; component 2 t=-2.8, *p*=0.014).

Looking at the PSP group only, component 1 correlated with subcortical volume (r=0.30, Δr^2^ = 0.08, t=2.4, *p*=0.038), but not frontal cortical thickness (r=0.03, t=0.24, *p*=0.96, Fig. 4C). Steiger's Z-test of the partial correlation coefficients did not find a significant difference (Steiger's Z=1.9, *p*=0.06). Component 2 did not correlate with either frontal cortical thickness (r=-0.20, t=-1.6, *p*=0.22), or subcortical grey matter volume (r=-0.19, t=-1.5, *p*=0.26, Fig. 4D).

We then tested whether changes in brain state occupancy were driven by randomisation of the network and connectivity changes in subcortical and cortical regions in response to focal atrophy. Component 2 scores correlated negatively with clustering coefficient (r=-0.23, t=-2.6, *p*=0.012, Fig. 4F, replicated between thresholds 2%–10%) suggesting increasing randomness of the brain's network topology. No significant correlation was found with path length (r=-0.14, t=-1.4, *p*=0.14). The relationship between weighted degree and component 2 differed between people with PSP and controls in both cortical and subcortical regions (cortical F=4, Δr^2^ = 0.03, *p*=0.047; subcortical F=4, Δr^2^ = 0.03, *p*=0.048) and was steeper in participants with PSP (cortical r=-0.31, t=-2.5 *p*=0.014; subcortical r=-0.34 t=-2.8 *p*=0.006) than in controls (cortical r=0, t=-0.02 *p*=0.98; subcortical r=-0.06 t=-0.44 *p*=0.66). No significant relationships were found with component 1.

Despite the relationships between network dynamics, focal atrophy and topological changes, we found no significant difference by clinical phenotype (component 1 F=3.4 *p*=0.083; component 2 F=0.57, *p*=0.82).

## 4 Discussion

The principal results of this study are that (i) the exemplar tauopathy of PSP changes network dynamics, with a higher proportion of time spent in frontoparietal activation states; (ii) these changes in network dynamics are related to complexity as measured by multi-scale entropy, (iii) the changes in network dynamics correlated with clinical severity and regional atrophy; and (iv) altered network dynamics occur in the context of widespread changes to network topology. The effect of PSP phenotypic variance is expressed in terms of the relationship between network dynamics, clinical severity and focal atrophy, in cortical *versus* subcortical regions.

In two independent datasets, people with PSP spent more time in states whose spatial distributions mirror executive control networks. Given that in health, occupancy of networks associated with higher order cognition correlates positively with cognitive function (Vidaurre et al., 2017), these results may seem surprising. Time in these networks did not correlate with frontal atrophy but did show a negative relationship with clustering coefficient and weighted degree only in PSP. This suggests a loss of small-world properties towards greater network randomness (Bassett and Bullmore, 2016; Watts and Strogatz, 1998), with occupancy of a remodeled and more random network no longer relating to its effective functioning.

PSP causes severe disruption to connectivity between the subcortex/brainstem and cortical regions (Brown et al., 2017; Whitwell et al., 2011), changes that may account for our finding of reduced time in states with activity in subcortical regions, which correlated with subcortical atrophy. So, why do participants with PSP spend more time in executive control networks and less time in states representing the default mode network with negative activations in regions of the task-positive network? Regional structural network properties influence brain state transitions, with access to frontoparietal cognitive control networks depending on nodes within weakly connected regions (Gu et al., 2015). Key nodes in a dysfunctional random network may no longer effectively determine state transitions, causing altered network dynamics in regions remote from the primary pathology. Specifically, the results shed light on the interplay in disease between atrophy, remodeling of network topology and network dynamics.

We found that network dynamics in PSP varied by disease severity, both in terms of relationship to atrophy and to the PSPRS: the latter is sensitive to disease progression and predicts survival (Bang et al., 2016; Golbe and Ohman-Strickland, 2007). We did not find that this dependency translated into differences between PSP phenotypes, although our study was powered to only detect large subgroup differences. Nonetheless, given that the intrinsic network architecture of the brain present at rest shapes that seen during tasks (Cole et al., 2014; Smith et al., 2009), and that network structure predicts behavioral traits (Arbabshirani et al., 2017; Beaty et al., 2018; Meer et al., 2020; Rosenberg et al., 2016), we hypothesize that the observed changes in brain state transitions in PSP underpin cognitive symptoms of the disease.

We have shown that measuring complexity provides a complementary method to assess temporal dynamics in disease. In the CCPP dataset we found reduced complexity in PSP, with the greatest differences seen at the highest scale, indicating a true reduction in complexity and not randomness. This result was not seen in PROSPECT-MR, perhaps because these images were acquired with fewer time points. We found that complexity correlated with both switching rate and fractional occupancy component, suggesting that changes in global signal influence network dynamics in PSP. This raises the possibility of additional aetiological factors to those outlined above, including the profound neurotransmitter deficits which occur early in PSP (Murley and Rowe, 2018) and alter global fMRI signal (Turchi et al., 2018) and dynamic connectivity (Shine et al., 2018) in health.

There has been controversy as to whether variability in dynamic functional connectivity in task-free fMRI is more than motion (Laumann et al., 2017)*.* We used stringent exclusion criteria to limit the impact of movement artefact. A key advantage of this study is that results are replicated in two datasets, an important part of the solution to non-generalizable results in neuroimaging due to low statistical power and analytic flexibility (Poldrack et al., 2017). Our approach did however result in significant numbers of exclusions, particularly from our CCPP dataset (due to the longer time series). It may be that these excluded participants have distinct clinical phenotypes, and therefore our conclusions would not apply to all individuals with PSP. Modelling network dynamics via an HMM requires forced choices in analysis, notably the number of states inferred. If brain states consist of a hierarchy of structures (Vidaurre et al., 2018) it is likely that HMMs can provide multiple related solutions. Indeed, this may account for some of the anatomical differences observed in our two datasets. We believe that this challenge is best tackled by independent replication and relating findings to clinical scores.

## 5 Conclusions

The quantification of the dynamics of large-scale resting state networks offers an intermediate phenotype with which to understand clinical syndrome. Through hidden Markov modelling we have shown that changes in network dynamics relate to neural signal complexity and to phenotypic variance in progressive supranuclear palsy. Our methodology demonstrates how abnormalities in regional atrophy and topological changes correlate with brain state transitions, and provides a means to directly test the causes and consequences of altered network dynamics.

## Verification

We verify that this work has not been published before, is not under consideration for publication elsewhere, and if accepted will not be published elsewhere in the same form. Submission for publication is approved by all authors.

## Disclosure statement

J.B.R. serves as an associate editor to Brain and is a nonremunerated trustee of the Guarantors of Brain and the PSP Association (UK). He provides consultancy to Asceneuron, Biogen, and UCB and has research grants from AZ‐Medimmune, Janssen, and Lilly as industry partners in the Dementias Platform UK. M.T.H received payment for Advisory Board attendance/consultancy for Biogen, Roche, CuraSen Therapeutics, Evidera, Manus Neurodynamica and the MJFF Digital Health Assessment Board. M.T.H is a co-applicant on a patent application related to smartphone predictions in Parkinson's (PCT/GB2019/052522) pending. All other authors did not declare any funding sources that directly contributed to this study.

## Acknowledgements

The study was co-funded by the National Institute for Health Research (NIHR) Biomedical Research Centre at Cambridge University Hospitals NHS Foundation Trust and the University of Cambridge (BRC-1215-20014), the 10.13039/501100019544Cambridge Centre for Parkinson-plus (RG95450); the Wellcome Trust (220258); the 10.13039/501100004282Evelyn Trust (17/09); National Institute for Health Research (ACF-2018-14-016); the PSP Association UK (RG78738); Parkinson's UK; NIHR Oxford Biomedical Research Centre; NIHR Oxford Health Clinical Research Facility; University of Oxford; Michael J Fox Foundation; NIHR University College London Hospitals (UCLH) Biomedical Research Centre; the Edmond J. Safra Philanthropic Foundation; and the NIHR UCLH Clinical Research Facility. The views expressed are those of the authors and not necessarily those of the NIHR or the Department of Health and Social Care.

## CRediT authorship contribution statement

**David J Whiteside:** Formal analysis, Writing – original draft, Writing – review & editing, Visualization. **P. Simon Jones:** Data curation. **Boyd C P Ghosh:** Investigation. **Ian Coyle-Gilchrist:** Investigation, Writing – review & editing. **Alexander Gerhard:** Resources, Project administration. **Michele T. Hu:** Resources, Project administration, Writing – review & editing. **Johannes C Klein:** Resources, Project administration. **P. Nigel Leigh:** Resources, Project administration. **Alistair Church:** Resources, Project administration. **David J Burn:** Resources, Project administration. **Huw R Morris:** Resources, Project administration, Funding acquisition. **James B Rowe:** Conceptualization, Methodology, Investigation, Resources, Writing – review & editing, Supervision, Project administration, Funding acquisition. **Timothy Rittman:** Conceptualization, Methodology, Investigation, Resources, Writing – review & editing, Supervision, Project administration.

## Appendix Supplementary materials

Image, application 1

## References

[bib0001] Achard S., Bullmore E. (2007). Efficiency and cost of economical brain functional networks. PLoS Comput Biol.

[bib0002] Ahmed Z., Cooper J., Murray T.K., Garn K., McNaughton E., Clarke H., Parhizkar S., Ward M.A., Cavallini A., Jackson S., Bose S., Clavaguera F., Tolnay M., Lavenir I., Goedert M., Hutton M.L., O'Neill M.J. (2014). A novel in vivo model of tau propagation with rapid and progressive neurofibrillary tangle pathology: the pattern of spread is determined by connectivity, not proximity. Acta Neuropathol.

[bib0003] Arbabshirani M.R., Plis S., Sui J., Calhoun V.D. (2017). Single subject prediction of brain disorders in neuroimaging: Promises and pitfalls. NeuroImage.

[bib0004] Bang J., Lobach I.V., Lang A.E., Grossman M., Knopman D.S., Miller B.L., Schneider L.S., Doody R.S., Lees A., Gold M., Morimoto B.H., Boxer A.L., Investigators A.L. (2016). Predicting disease progression in progressive supranuclear palsy in multicenter clinical trials. Parkinsonism Relat Disord.

[bib0005] Bassett D.S., Bullmore E.T. (2016). Small-world brain networks revisited. Neurosci.

[bib0006] Beaty R.E., Kenett Y.N., Christensen A.P., Rosenberg M.D., Benedek M., Chen Q., Fink A., Qiu J., Kwapil T.R., Kane M.J., Silvia P.J. (2018). Robust prediction of individual creative ability from brain functional connectivity. Proc Natl Acad Sci.

[bib0007] Brown J.A., Hua A.Y., Trujillo A., Attygalle S., Binney R.J., Spina S., Lee S.E., Kramer J.H., Miller B.L., Rosen H.J., Boxer A.L., Seeley W.W. (2017). Advancing functional dysconnectivity and atrophy in progressive supranuclear palsy. NeuroImage: Clinical.

[bib0008] Burrell J.R., Hodges J.R., Rowe J.B. (2014). Cognition in corticobasal syndrome and progressive supranuclear palsy: A review. Movement Disorders.

[bib0009] Calhoun V.D., Miller R., Pearlson G., Adalı T. (2014). The chronnectome: time-varying connectivity networks as the next frontier in fMRI data discovery. Neuron.

[bib0010] Clavaguera F., Bolmont T., Crowther R.A., Abramowski D., Frank S., Probst A., Fraser G., Stalder A.K., Beibel M., Staufenbiel M., Jucker M., Goedert M., Tolnay M. (2009). Transmission and spreading of tauopathy in transgenic mouse brain. Nat Cell Biol.

[bib0011] Cole Michael W., Bassett Danielle S., Power Jonathan D., Braver Todd S., Petersen Steven E. (2014). Intrinsic and task-evoked network architectures of the human brain. Neuron.

[bib0012] Costa M., Goldberger A.L., Peng C.K. (2005). Multiscale entropy analysis of biological signals. Phys Rev E Stat Nonlin Soft Matter Phys.

[bib0013] Dale A.M., Fischl B., Sereno M.I. (1999). Cortical surface-based analysis. I. segmentation and surface reconstruction. Neuroimage.

[bib0014] Deco G., Kringelbach M.L., Jirsa V.K., Ritter P. (2017). The dynamics of resting fluctuations in the brain: metastability and its dynamical cortical core. Sci Rep.

[bib0015] Eldar E., Cohen J.D., Niv Y. (2013). The effects of neural gain on attention and learning. Nat Neurosci.

[bib0016] Fan L., Li H., Zhuo J., Zhang Y., Wang J., Chen L., Yang Z., Chu C., Xie S., Laird A.R., Fox P.T., Eickhoff S.B., Yu C., Jiang T. (2016). The human brainnetome atlas: a new brain atlas based on connectional architecture. Cereb Cortex.

[bib0017] Friston K., Breakspear M., Deco G. (2012). Perception and self-organized instability. Front Comput Neurosci.

[bib0018] Gardner R.C., Boxer A.L., Trujillo A., Mirsky J.B., Guo C.C., Gennatas E.D., Heuer H.W., Fine E., Zhou J., Kramer J.H., Miller B.L., Seeley W.W. (2013). Intrinsic connectivity network disruption in progressive supranuclear palsy. Ann Neurol.

[bib0019] Ghanbari Y., Bloy L., Christopher Edgar J., Blaskey L., Verma R., Roberts T.P.L. (2015). Joint analysis of band-specific functional connectivity and signal complexity in autism. J Autism Dev Disord.

[bib0020] Golbe L.I., Ohman-Strickland P.A. (2007). A clinical rating scale for progressive supranuclear palsy. Brain.

[bib0021] Grandy T.H., Garrett D.D., Schmiedek F., Werkle-Bergner M. (2016). On the estimation of brain signal entropy from sparse neuroimaging data. Sci Rep.

[bib0022] Gu S., Pasqualetti F., Cieslak M., Telesford Q.K., Yu A.B., Kahn A.E., Medaglia J.D., Vettel J.M., Miller M.B., Grafton S.T., Bassett D.S. (2015). Controllability of structural brain networks. Nat Commun.

[bib0023] Hagberg, A., Swart, P., S Chult, D., 2008. Exploring network structure, dynamics, and function using networkx. United States.

[bib0024] Hindriks R., Adhikari M.H., Murayama Y., Ganzetti M., Mantini D., Logothetis N.K., Deco G. (2016). Can sliding-window correlations reveal dynamic functional connectivity in resting-state fMRI?. NeuroImage.

[bib0025] Höglinger G.U., Respondek G., Stamelou M., Kurz C., Josephs K.A., Lang A.E., Mollenhauer B., Müller U., Nilsson C., Whitwell J.L., Arzberger T., Englund E., Gelpi E., Giese A., Irwin D.J., Meissner W.G., Pantelyat A., Rajput A., van Swieten J.C., Troakes C., Antonini A., Bhatia K.P., Bordelon Y., Compta Y., Corvol J.-C., Colosimo C., Dickson D.W., Dodel R., Ferguson L., Grossman M., Kassubek J., Krismer F., Levin J., Lorenzl S., Morris H.R., Nestor P., Oertel W.H., Poewe W., Rabinovici G., Rowe J.B., Schellenberg G.D., Seppi K., van Eimeren T., Wenning G.K., Boxer A.L., Golbe L.I., Litvan I., Society-endorsed, P.S.P.S.G. Movement Disorder (2017). Clinical diagnosis of progressive supranuclear palsy: The movement disorder society criteria. Mov Disord.

[bib0026] Honey C.J., Kötter R., Breakspear M., Sporns O. (2007). Network structure of cerebral cortex shapes functional connectivity on multiple time scales. Proc Nat Acad Sci.

[bib0027] Hsieh S., Schubert S., Hoon C., Mioshi E., Hodges J.R. (2013). Validation of the addenbrooke's cognitive examination III in frontotemporal dementia and alzheimer's Disease. Dement Geriatr Cogn Disord.

[bib0028] Iglesias J.E., Van Leemput K., Bhatt P., Casillas C., Dutt S., Schuff N., Truran-Sacrey D., Boxer A., Fischl B. (2015). Bayesian segmentation of brainstem structures in MRI. Neuroimage.

[bib0029] Jabbari E., Holland N., Chelban V., Jones P.S., Lamb R., Rawlinson C., Guo T., Costantini A.A., Tan M.M.X., Heslegrave A.J., Roncaroli F., Klein J.C., Ansorge O., Allinson K.S.J., Jaunmuktane Z., Holton J.L., Revesz T., Warner T.T., Lees A.J., Zetterberg H., Russell L.L., Bocchetta M., Rohrer J.D., Williams N.M., Grosset D.G., Burn D.J., Pavese N., Gerhard A., Kobylecki C., Leigh P.N., Church A., Hu M.T.M., Woodside J., Houlden H., Rowe J.B., Morris H.R. (2019). Diagnosis across the spectrum of progressive supranuclear palsy and corticobasal syndrome. JAMA Neurology.

[bib0030] Kaalund S.S., Passamonti L., Allinson K.S.J., Murley A.G., Robbins T.W., Spillantini M.G., Rowe J.B. (2020). Locus coeruleus pathology in progressive supranuclear palsy, and its relation to disease severity. Acta Neuropathologica Communications.

[bib0031] Kovacs G.G., Lukic M.J., Irwin D.J., Arzberger T., Respondek G., Lee E.B., Coughlin D., Giese A., Grossman M., Kurz C., McMillan C.T., Gelpi E., Compta Y., van Swieten J.C., Laat L.D., Troakes C., Al-Sarraj S., Robinson J.L., Roeber S., Xie S.X., Lee V.M.Y., Trojanowski J.Q., Höglinger G.U. (2020). Distribution patterns of tau pathology in progressive supranuclear palsy. Acta Neuropathol.

[bib0032] Laumann T.O., Snyder A.Z., Mitra A., Gordon E.M., Gratton C., Adeyemo B., Gilmore A.W., Nelson S.M., Berg J.J., Greene D.J., McCarthy J.E., Tagliazucchi E., Laufs H., Schlaggar B.L., Dosenbach N.U.F., Petersen S.E. (2017). On the Stability of BOLD fMRI Correlations. Cereb Cortex.

[bib0033] Ling H., de Silva R., Massey L.A., Courtney R., Hondhamuni G., Bajaj N., Lowe J., Holton J.L., Lees A., Revesz T. (2014). Characteristics of progressive supranuclear palsy presenting with corticobasal syndrome: a cortical variant. Neuropathol Appl Neurobiol.

[bib0034] McDonough I.M., Nashiro K. (2014). Network complexity as a measure of information processing across resting-state networks: evidence from the Human Connectome Project. Front Hum Neurosci.

[bib0035] McIntosh A.R., Vakorin V., Kovacevic N., Wang H., Diaconescu A., Protzner A.B. (2014). Spatiotemporal dependency of age-related changes in brain signal variability. Cereb Cortex.

[bib0036] Meer J.N.v.d., Breakspear M., Chang L.J., Sonkusare S., Cocchi L. (2020). Movie viewing elicits rich and reliable brain state dynamics. Nature Communications.

[bib0037] Mioshi E., Dawson K., Mitchell J., Arnold R., Hodges J.R. (2006). The addenbrooke's cognitive examination revised (ACE-R): a brief cognitive test battery for dementia screening. Int J Geriatr Psychiatr.

[bib0038] Murley A.G., Rowe J.B. (2018). Neurotransmitter deficits from frontotemporal lobar degeneration. Brain.

[bib0039] Nickerson L.D., Smith S.M., Öngür D., Beckmann C.F. (2017). Using dual regression to investigate network shape and amplitude in functional connectivity analyses. Front Neurosci.

[bib0040] Patel A.X., Kundu P., Rubinov M., Jones P.S., Vértes P.E., Ersche K.D., Suckling J., Bullmore E.T. (2014). A wavelet method for modeling and despiking motion artifacts from resting-state fMRI time series. NeuroImage.

[bib0041] Pincus S.M., Goldberger A.L. (1994). Physiological time-series analysis: what does regularity quantify?. Am J Physiol.

[bib0042] Poldrack R.A., Baker C.I., Durnez J., Gorgolewski K.J., Matthews P.M., Munafò M.R., Nichols T.E., Poline J.-B., Vul E., Yarkoni T. (2017). Scanning the horizon: towards transparent and reproducible neuroimaging research. Nature Reviews Neuroscience.

[bib0043] Power J.D., Barnes K.A., Snyder A.Z., Schlaggar B.L., Petersen S.E. (2012). Spurious but systematic correlations in functional connectivity MRI networks arise from subject motion. NeuroImage.

[bib0044] Core Team R (2018).

[bib0045] Ray K., McKay D., Fox P., Riedel M., Uecker A., Beckmann C., Smith S., Laird A. (2013). ICA model order selection of task co-activation networks. Front Neurosci.

[bib0046] Richman J.S., Moorman J.R. (2000). Physiological time-series analysis using approximate entropy and sample entropy. Am J Physiol Heart Circ Physiol.

[bib0047] Rosenberg M.D., Finn E.S., Scheinost D., Papademetris X., Shen X., Constable R.T., Chun M.M. (2016). A neuromarker of sustained attention from whole-brain functional connectivity. Nat Neurosci.

[bib0048] Rosskopf J., Gorges M., Müller H.-P., Lulé D., Uttner I., Ludolph A.C., Pinkhardt E., Juengling F.D., Kassubek J. (2017). Intrinsic functional connectivity alterations in progressive supranuclear palsy: Differential effects in frontal cortex, motor, and midbrain networks. Movement Disorders.

[bib0049] Sakae N., Josephs K.A., Litvan I., Murray M.E., Duara R., Uitti R.J., Wszolek Z.K., Graff-Radford N.R., Dickson D.W. (2019). Neuropathologic basis of frontotemporal dementia in progressive supranuclear palsy. Movement Disorders.

[bib0050] Seeley W.W., Crawford R.K., Zhou J., Miller B.L., Greicius M.D. (2009). Neurodegenerative diseases target large-scale human brain networks. Neuron.

[bib0051] Shine J.M. (2019). Neuromodulatory Influences on Integration and Segregation in the Brain. Trends Cogn Sci.

[bib0052] Shine J.M., Breakspear M., Bell P.T., Ehgoetz Martens K.A., Shine R., Koyejo O., Sporns O., Poldrack R.A. (2019). Human cognition involves the dynamic integration of neural activity and neuromodulatory systems. Nat Neurosci.

[bib0053] Shine J.M., van den Brink R.L., Hernaus D., Nieuwenhuis S., Poldrack R.A. (2018). Catecholaminergic manipulation alters dynamic network topology across cognitive states. Netw Neurosci.

[bib0054] Smith R.X., Yan L., Wang D.J.J. (2014). Multiple time scale complexity analysis of resting state FMRI. Brain Imaging Behav.

[bib0055] Smith S.M., Fox P.T., Miller K.L., Glahn D.C., Fox P.M., Mackay C.E., Filippini N., Watkins K.E., Toro R., Laird A.R., Beckmann C.F. (2009).

[bib0056] Smyser C.D., Inder T.E., Shimony J.S., Hill J.E., Degnan A.J., Snyder A.Z., Neil J.J. (2010). Longitudinal analysis of neural network development in preterm infants. Cereb Cortex.

[bib0057] Steele J.C., Richardson J.C., Olszewski J. (1964). Progressive supranuclear palsy: a heterogeneous degeneration involving the brain stem, basal ganglia and cerebellum with vertical gaze and pseudobulbar palsy, nuchal dystonia and dementia. JAMA Neurology.

[bib0058] Tognoli E., Kelso J.A.S. (2014). The metastable brain. Neuron.

[bib0059] Tsuboi Y., Josephs K.A., Boeve B.F., Litvan I., Caselli R.J., Caviness J.N., Uitti R.J., Bott A.D., Dickson D.W. (2005). Increased tau burden in the cortices of progressive supranuclear palsy presenting with corticobasal syndrome. Movement Disorders.

[bib0060] Turchi J., Chang C., Ye F.Q., Russ B.E., Yu D.K., Cortes C.R., Monosov I.E., Duyn J.H., Leopold D.A. (2018). The basal forebrain regulates global resting-state fMRI fluctuations. Neuron.

[bib0061] Turkheimer F.E., Leech R., Expert P., Lord L.-D., Vernon A.C. (2015). The brain's code and its canonical computational motifs. From sensory cortex to the default mode network: A multi-scale model of brain function in health and disease. Neurosci Biobehav Rev.

[bib0062] Vidaurre D., Abeysuriya R., Becker R., Quinn A.J., Alfaro-Almagro F., Smith S.M., Woolrich M.W. (2018). Discovering dynamic brain networks from big data in rest and task. NeuroImage.

[bib0063] Vidaurre D., Llera A., Smith S.M., Woolrich M.W. (2021). Behavioural relevance of spontaneous, transient brain network interactions in fMRI. NeuroImage.

[bib0064] Vidaurre D., Smith S.M., Woolrich M.W. (2017). Brain network dynamics are hierarchically organized in time. Proc Nat Acad Sci.

[bib0065] Wang D.J.J., Jann K., Fan C., Qiao Y., Zang Y.-F., Lu H., Yang Y. (2018). Neurophysiological basis of multi-scale entropy of brain complexity and its relationship with functional connectivity. Front Neurosci.

[bib0066] Watts D.J., Strogatz S.H. (1998). Collective dynamics of ‘small-world’ networks. Nature.

[bib0067] Whitwell J.L., Avula R., Master A., Vemuri P., Senjem M.L., Jones D.T., Jack C.R., Jr., Josephs K.A. (2011). Disrupted thalamocortical connectivity in PSP: a resting-state fMRI, DTI, and VBM study. Parkinsonism Relat Disord.

[bib0068] Winkler A.M., Ridgway G.R., Webster M.A., Smith S.M., Nichols T.E. (2014). Permutation inference for the general linear model. NeuroImage.

[bib0069] Yang A.C., Huang C.-C., Yeh H.-L., Liu M.-E., Hong C.-J., Tu P.-C., Chen J.-F., Huang N.E., Peng C.-K., Lin C.-P., Tsai S.-J. (2013). Complexity of spontaneous BOLD activity in default mode network is correlated with cognitive function in normal male elderly: a multiscale entropy analysis. Neurobiol Aging.

[bib0070] Yang A.C., Tsai S.-J., Lin C.-P., Peng C.-K. (2018). A strategy to reduce bias of entropy estimates in resting-state fMRI signals. Front Neurosci.

