# Supplementary material for: Altered network stability in progressive supranuclear palsy
Source: Neurobiol Aging. 2021 Nov;107:109–17. doi: 10.1016/j.neurobiolaging.2021.07.007 (PMC8599965; doi:10.1016/j.neurobiolaging.2021.07.007)
Supplement: Supplementary file 1 [file mmc1.docx]

**Supplementary information**

**Analysis of network dynamics with age and sex-matched cohorts**

To ensure that our results were not biased by group differences in age and sex we repeated our analyses with a subset of controls and participants with PSP. For the CCPP cohort we removed participants outside the common support region for age, leaving 19 Controls and 22 participants with PSP. The remaining group was well matched for age (mean age (standard deviation); Controls=68.0 (6.2), PSP=69.1 (5.8), Welch’s two sample t-test t=-0.62 p=0.53) and for sex (female/male Controls 11/8, PSP 10/12, Chi-squared test χ=0.23 p=0.63).

There was no difference in switching rate between controls and participants with PSP (t=0.28, p=0.63). Three PCA components with eigenvalues greater than 1 explained 75% of the variance and were taken forward for further analysis. The first component was significantly more negative in PSP than controls (t=3.6, p=0.003). States with the highest positive loadings (states 5 and 7) and most negative loadings (states 1, 4 and 6) were unchanged from our primary analysis. Multi-scale entropy was again reduced in PSP (t=2.5, p=0.014).

For the PROSPECT cohort one healthy control and one participant with PSP were removed as outside the common support region for age. Given the residual significant differences in sex we performed further propensity matching with a caliper of 0.5, leaving 26 Controls and 26 participants with PSP. The remaining group was well matched for age (mean age (standard deviation); Controls=69.6 (5.7), PSP=71.8 (8.2), Welch’s two sample t-test t=-1.1 p=0.27) and for sex (female/male Controls 17/9, PSP 15/11, χ=0.08 p=0.78).

There was no difference in switching rates between controls and participants with PSP. Two components with eigenvalues greater than 1 explained 74% of the variance and were taken forward for further analysis. Component scores for both components were altered in PSP. Component 1 was increased in PSP (t=-2.8 p=0.015) with positive loadings from states 2, 3 and 4, and negative loadings for states 5 and 7. Component 2 was reduced in PSP (t=2.8 p=0.018), with State 8 the primary positive contributor and negative loadings for states 6 and 1. Although there was some difference from our primary analysis in the distribution of component loadings the pattern was unchanged, with increased time in PSP in frontoparietal networks and reduced time in subcortical networks. The reduction in multi-scale entropy in PSP was not significant (t=1.3, p=0.19)

**Supplementary table 1: Motion quality metrics for participants**

|  | **Control included** | **PSP included** | **t (p)** | **Control excluded** | **PSP excluded** |
| --- | --- | --- | --- | --- | --- |
| **Maximum spike percentage** | 9.8 (5.9) | 13.7 (7.9) | -3.2 (0.002) | 27.9 (13) | 41.6 (16) |
| **Median spike percentage** | 2.5 (1.3) | 2.5 (1.4) | -0.02 (0.98) | 6 (3.9) | 5.6 (4.3) |
| **Maximum framewise displacement** | 0.54 (0.45) | 1 (0.95) | -3.3 (0.001) | 2.1 (2.3) | 5.1 (7.1) |
| **Maximum dvars** | 7.3 (1.1) | 7.5 (1.1) | -0.84 (0.41) | 9.7 (0.8) | 10.4 (2.3) |

Values are mean (SD)

**Supplementary figure 1**


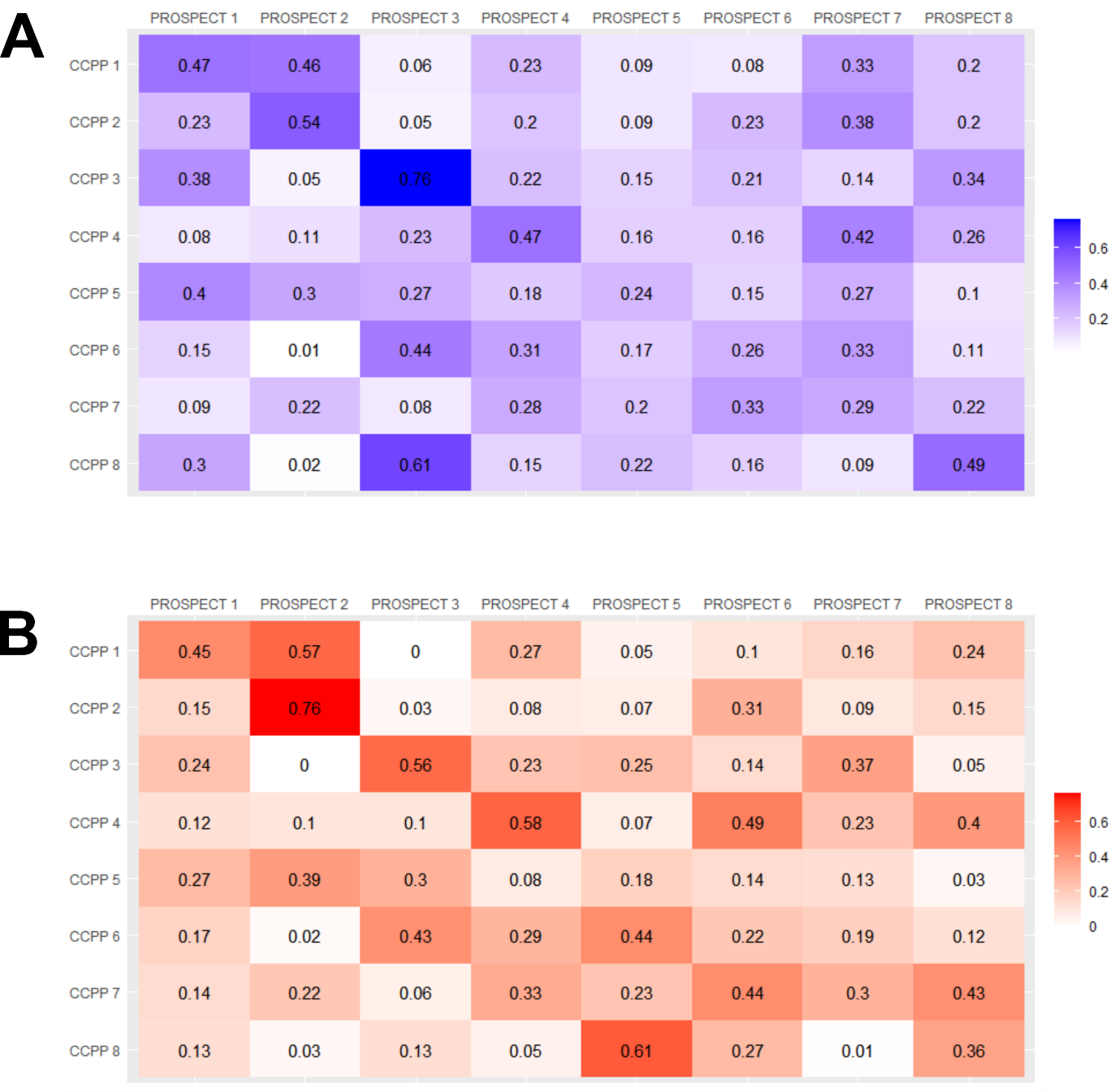


**Supplementary figure 1:** Dice coefficients between binarised A) negative and B) positive CCPP and PROSPECT-MR states
